# Post COVID-19 rhinocerebral mucormycosis: a case report and literature review

**DOI:** 10.11604/pamj.2022.42.202.33690

**Published:** 2022-07-14

**Authors:** Amel El Korbi, Salma Bhar, Emna Bergaoui, Khaled Harrathi, Jamel Koubaa

**Affiliations:** 1 Department of Ear, Nose, and Throat (ENT), Fattouma Bourguiba Teaching Hospital, Monastir, Tunisia,; 2 Research Unit, Quality and Security of Care, University of Monastir, Monastir, Tunisia

**Keywords:** COVID-19, rhinocerebral, mucormycosis, diabetes, case report

## Abstract

The pandemic of coronavirus disease 2019 (COVID-19) still remains on an upsurge trend. The second and the waves of this disease have led to panic in many countries, and some parts of the world suffering from the fourth wave. In the midst of this pandemic, COVID-19 patients are acquiring secondary infections such as mucormycosis also known as “black fungus disease”. Mucormycosis is a serious, but rare opportunistic fungal infection that spreads rapidly, and hence prompt diagnosis and treatment are necessary to avoid mortality and morbidity rate. We report in this paper, a case of a diabetic patient who presented with bilateral nasal obstruction, anosmia, and frontal headache diagnosed with rhinocerebral mucormycosis developing after COVID-19 infection with a favorable outcome after a medico-surgical treatment. Through this case, we aim to aware patricians of this possible association and the importance of early diagnosis to optimize treatment outcomes.

## Introduction

Mucormycosis is a rare life-threatening invasive fungal infection caused by mold fungi of the genus *Mucor, Rhizopus, Rhizomucor* and *Absidia* which belong to the Mucorales order of the zygomycetes class [1]. It was possibly first described by Friedrich Küchenmeister in 1855 [1]. The most common type was *Rhizopus oryzae* responsible for approximately 60% of mucormycosis cases in humans; and 90% of the rhinocerebral form [2]. Over this pandemic of coronavirus disease (COVID-19), we observed a worldwide increasing mucormycosis in COVID-19 infected patients. It is frequently observed in immunosuppressed patients, particularly with diabetes, hematological malignancy, and immunosuppressive and corticosteroid therapy. It is rarely seen in healthy individuals. Here we report a case of rhinocerebral mucormycosis developed after COVID-19 infection in a diabetic patient who presented with bilateral nasal obstruction, anosmia, and frontal headache. Through this case, we aim to aware patricians of this possible association and the importance of early diagnosis to optimize treatment outcomes.

## Patient and observation

**Patient information:** a 62-year-old male patient with a history of type 2 diabetes, hypertension and coronary artery disease was referred to our department for persistent nasal obstruction and frontal headache.

**Figure 1:**
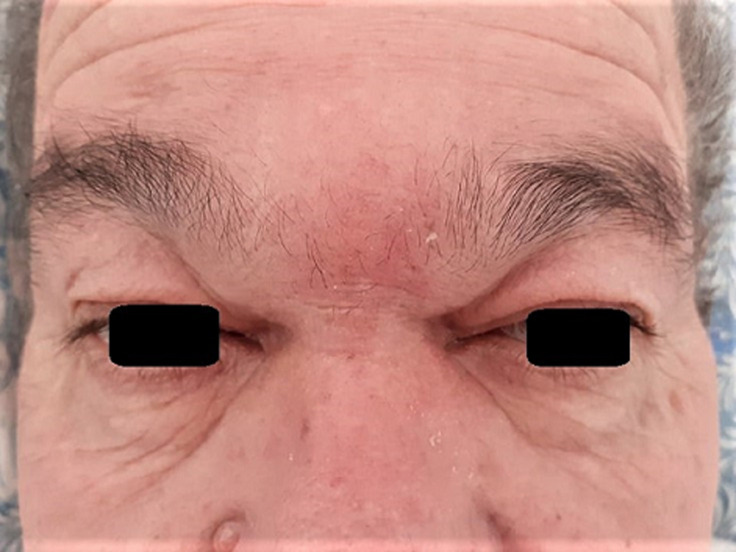
frontal swelling with edema of the upper right eyelid

**Figure 2:**
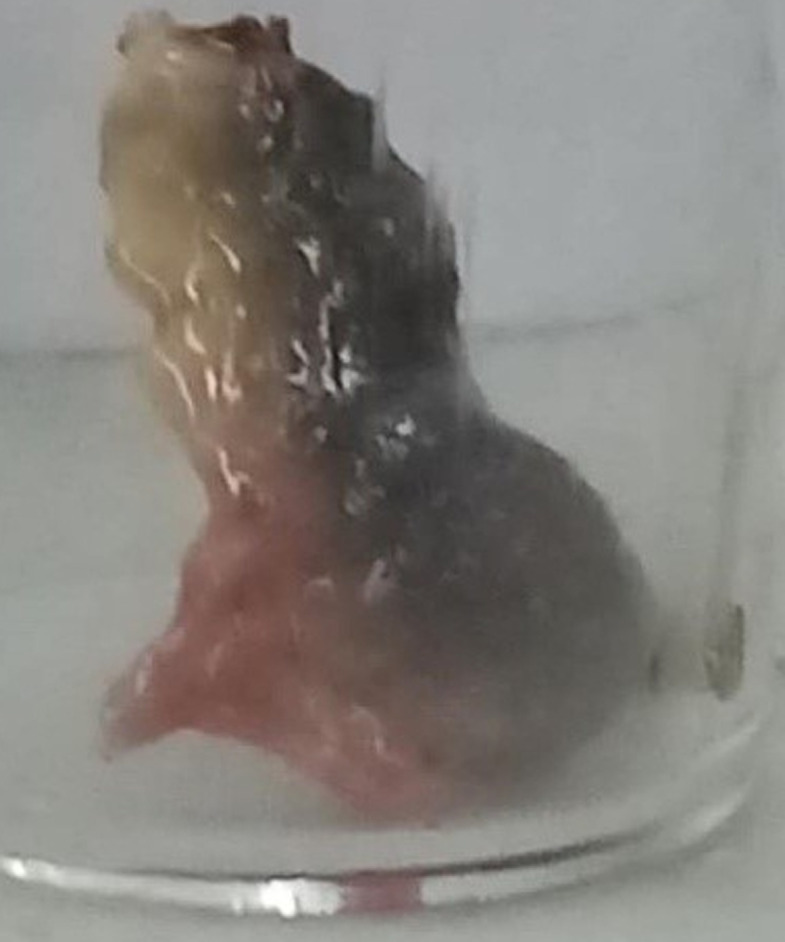
necrotic right middle turbinate

**Clinical findings:** clinical exam found a frontal swelling associated with edema of the upper right eyelid (Figure 1). Nasal endoscopy showed the presence of pus in the middle meatuses and necrotic appearance of the middle turbinates (Figure 2).

**Timeline of current episode:** in fact, the patient was hospitalized for COVID-19 infection the third of January 2021 then he was referred to an intensive care unit where he received a high-flow oxygen for thirteen days combined with corticosteroid therapy, then the patient was transferred to the pandemic ward and discharged the 29^th^ of January after a stay of 14 days. The patient presented since leaving the hospital with bilateral nasal obstruction, anosmia and frontal headaches. He consulted an otolaryngologist doctor who referred the patient to our department on the sixth of March 2021.

**Figure 3:**
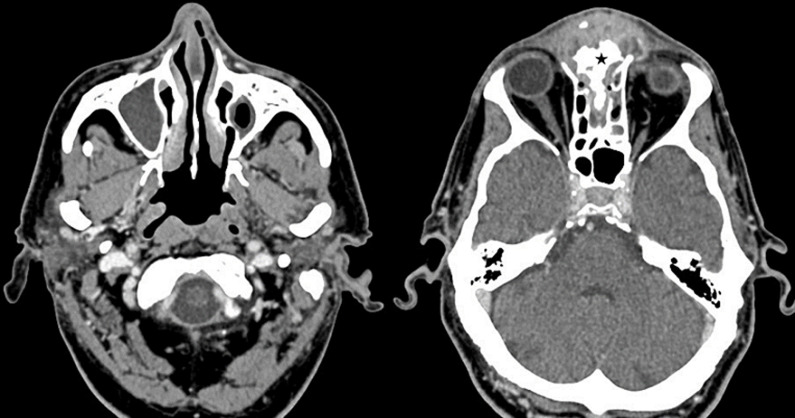
axial paranasal sinus computed tomography showing a pansinusitis with frontal osteitis and bone sequester (black asterix) surrounded by a soft tissue edema

**Diagnostic assessment:** biopsy of the middle turbinate and microbiological sampling were performed, with a presumed diagnosis of mucormycosis. Paranasal sinus computed tomography (CT) revealed pansinusitis with frontal osteitis and bone sequester surrounded by soft tissue edema (Figure 3). A cerebral magnetic resonance imaging (MRI) showed a frontal meningeal contrast enhancement without intracranial collection.

**Figure 4:**
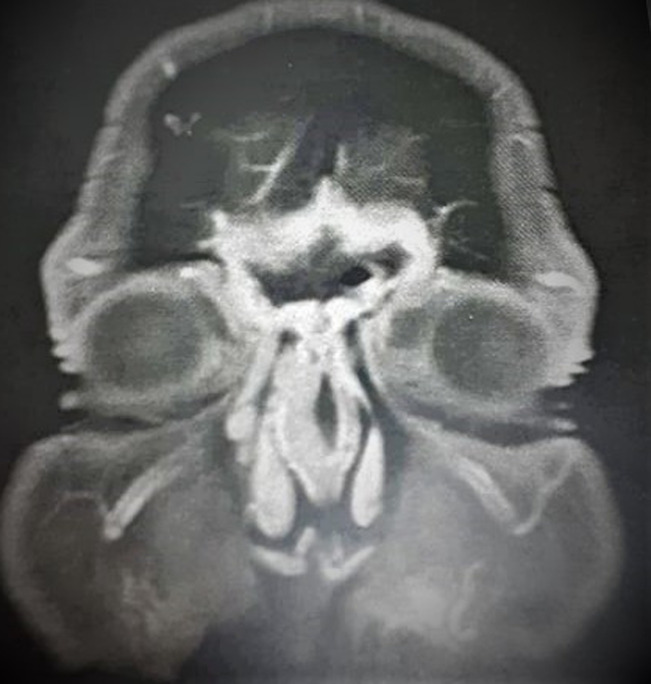
cerebral magnetic resonance imaging showing a bifrontal meningeal contrast enhancement without intracranial invasion

**Diagnosis:** the diagnosis of mucormycosis was confirmed on histopathologic and direct microscopy examination of the specimen that showed typical Mucorales hyphae. Whereas, culture was not contributed in the specie identification.

**Therapeutic interventions:** intravenous liposomal amphotericin B at the dose of 5 mg/kg/day was started early and debridement of the infection site was done. The antifungal treatment was extended for two months.

**Follow-up and outcome of interventions:** during the hospitalization, the patient refused to take treatment for three days explained to side effects such as hypokalemia and vomiting that were corrected by an intravenous supplementation, and the antifungal was reintroduced. After one month, an improvement of nasal symptoms and headache with the regression of the frontal swelling were observed. However, the post-therapeutic MRI showed stability of the radiological findings. The patient was discharged after a clinical improvement. After a follow-up of seven months, we noted the persistence of the frontal swelling without any other sign. Nasal endoscopy did not show any sign of mucosa necrosis.

**Patient perspective:** globally, despite the problem of antifungals intolerance, the patient showed satisfaction with his pathology´s care, and he adhered perfectly to the follow-up.

**Informed consent:** written informed consent was obtained from the patient for publication of this case report. A copy of the written consent is available for review.

## Discussion

Two thousand twenty is a memorable, devastating year for global health, as an uncommon virus raced worldwide, emerging rapidly. It is one of the top killers, laying bare the inadequacies of the health systems. Today, worldwide health services are struggling to tackle COVID-19 and provide people with vital care. As the global COVID-19 pandemic enters the third year, countries around the world are racing to vaccinate their populations as novel variants emerge. In the midst of this pandemic; the COVID-19 patients are acquiring secondary infections such as mucormycosis also known as “black fungus disease” [1]. Habitually, mucormycosis is a rare, opportunistic life-threatening fungal disease. However, with COVID-19 infection, we have seen an explosion of cases. In fact, in March 2021, only 41 cases of mucormycosis associated with COVID-19 infection have been reported worldwide and 70% were from India [1], contrasting with more than 47,000 cases reported in India from May to July 2021 [3]. Rhino-orbital and rhino-orbito-cerebral forms were the most common presentations. Patel *et al*. [4], reports in their multicenter epidemiologic study a predominance of rhino-orbital mucormycosis associated with COVID-19 with 62.6% of cases followed by rhino-orbito-cerebral in 23.5% and pulmonary form in 8.6% [3]. The main mode of transmission of mucormycosis is the inhalation of spores, ingestion of contaminated food and inoculation of the fungi into abrasions or cuts on the skin [1]. In healthy people, mononuclear and polymorphonuclear phagocytes eliminate fungal spores and hyphae [5], which is not the case of immunocompromised patients. A recent meta-analysis of 600 publications reported as major risk factors of mucormycosis: diabetes mellitus, trauma, hematological malignancies, diabetic ketoacidosis, neutropenia and prolonged corticosteroid therapy [6]. In the particular context of COVID-19 in infected patients, we observed an immune dysregulation with reduced numbers of T lymphocytes, CD4+T, and CD8+T cells that compromise innate immunity and enhance the risk of invasive fungal occurrence [7]. This risk is majored by the use of corticosteroid medications that magnify the effect of hyperglycemia in an eventual preexisting diabetic, and mechanical ventilation that contribute to inhalation of spores in immunocompromised individuals [1,8]. Moreover, cytokines released during COVID-19 increases intracellular iron and leakage of iron into the circulation, leading to high ferritin levels that permit the growth of Mucorales [3].

In our case, diabetes and corticosteroids firstly prescribed to manage the COVID-19 pulmonary disease were the risk factors of mucormycosis coinfection. Clinical manifestations depend on the extension of a disease. The symptoms of rhino-orbital mucormycosis are similar to complicated rhinosinusitis with nasal obstruction, headache, facial pain, bloody nasal discharge, swelling of periorbital tissues, orbital cellulitis, chemosis, proptosis, blurry vision, and local eschar that is explained by a propensity of blood vessels invasion by Mucorales leading to thrombosis, ischemia and tissue necrosis [9-11]. Intracranial involvement may manifests neurological signs or altered mental status [9]. Mucormycosis is usually suspected based on results of direct microscopy of clinical specimens and confirmed the diagnosis on histological features when showing tissue invasion by non-pigmented hyphae in tissue sections stained with haematoxylin-eosin [12]. Culture are recommended for genus and species identification, not for diagnosis [12]. In the study of Patel *et al*. [4], mucormycosis diagnosis was made by direct microscopy in 82.6% of patients and histopathology in 90.5%. Culture identified the etiologic pathogen in 4<8.1% of cases [4]. In our case, the diagnosis was made on microscopy and histopathology constitutions, however the culture was not able to identify the specie of the Mucorales. Refer to the 2019 global guidelines for the diagnosis and treatment of mucormycosis published by the European Confederation of Medical Mycology in cooperation with the Mycoses Study Group Education and research Consortium, the treatment of mucormycosis is based on early and complete surgical debridement of the infected area associated with a systemic antifungal drugs [12]. Mucorales are resistant to many antifungal agents. The most active ones are liposomal amphotericin B (LAB) and triazoles (isavuconazole and posaconazole), thus they are considered as first-line therapy in mucomycosis [5]. The recommended dose of LAB is 5 mg/kg per day in cases of non-central nervous system involvement [12]. Otherwise, a prescription of high dose of LAB at 10 mg/kg/day could be supported in cases of intracranial invasion [12]. Triazoles are reported to be more frequently used in patients with COVID-19 associated mucormycosis than those with isolated mucormycosis [4]. The duration of treatment with active antifungal agents has not been determined, in general weeks to months of therapy are given [12,13]. The mortality rate of mucormycosis is about 50% [1]. In their study, comparing mucormycosis associated with COVID-19 to isolated mucormycosis, Patel *et al*. [4] found similar mortality rates in both groups. In our case, we observed favorable outcomes after a follow-up of seven months.

## Conclusion

Through our case, we highlight the possibility of invasive secondary fungal infections in patients with COVID-19 infection. Physicians should be aware of early symptoms of mucormycosis to enable prompt diagnosis and adequate treatment to avoid as possible as can bad outcomes.
